# The Role of Soluble Suppression of Tumorigenicity-2 and Galectin-3 in Heart Failure With Preserved Ejection Fraction: A Scoping Review of the Literature

**DOI:** 10.31083/RCM51830

**Published:** 2026-09-20

**Authors:** Konstantina Ntalekou, Panagiotis Iliakis, Nikitas Katsillis, Nikolaos Patsourakos, Dimitrios Tsiachris, Dimitrios Farmakis, Polykarpos Christos Patsalis, Christina Chrysohoou, Konstantinos Tsioufis

**Affiliations:** ^1^First Department of Cardiology, School of Medicine, National and Kapodistrian University of Athens, Hippokration General Hospital, 11527 Athens, Greece; ^2^Cardiology Department, “Tzaneio” General Hospital of Piraeus, 18536 Piraeus, Greece; ^3^Division of Cardiology, Angiology and Internal Emergency Medicine, Department of Medicine, Knappschaft Kliniken University Hospital Bochum, Ruhr University Bochum, 44892 Bochum, Germany; ^4^Department of Cardiology, Attikon University Hospital, National and Kapodistrian University of Athens, 12462 Athens, Greece

**Keywords:** biomarkers, diagnosis, Gal-3, heart failure, preserved ejection fraction, prognosis, sST2

## Abstract

Heart failure with preserved ejection fraction (HFpEF) represents a heterogeneous clinical syndrome characterized by complex pathophysiological mechanisms and substantial diagnostic and prognostic challenges. Recently, circulating biomarkers have emerged as potential tools for improving risk stratification and disease characterization in this population. Among these biomarkers, soluble suppression of tumorigenicity-2 (sST2) and galectin-3 (Gal-3) have gained particular attention due to their association with myocardial fibrosis, inflammation, and adverse cardiac remodeling. This scoping review aimed to evaluate the current evidence on the diagnostic and prognostic roles of sST2 and Gal-3 in patients with HFpEF. A literature search was conducted in PubMed and ScienceDirect to identify studies investigating the clinical significance of these biomarkers in HFpEF. Titles and abstracts were screened, and eligible studies were assessed through full-text review according to predefined inclusion criteria. Ultimately, 11 studies were included in the qualitative synthesis. Evidence from the available literature suggests that both sST2 and Gal-3 are associated with markers of myocardial fibrosis, inflammation, and disease severity in HFpEF. Elevated circulating levels of these biomarkers have been linked to poorer clinical outcomes, including an increased risk of hospitalization and mortality. However, variability across studies in study populations, biomarker thresholds, and methodological approaches limits the direct translation of these findings into routine clinical practice. Overall, sST2 and Gal-3 appear to hold promise as complementary biomarkers for risk stratification in HFpEF. Nonetheless, further large-scale prospective studies are needed to clarify the clinical utility of these biomarkers and determine the subsequent integration into multimarker strategies for the management of patients with HFpEF.

## 1. Introduction

Heart failure (HF) with preserved ejection fraction (HFpEF) is a significant clinical syndrome with increasing prevalence, defined by signs and symptoms of heart failure, a left ventricular ejection fraction (LVEF) ≥50%, and evidence of structural and/or functional abnormalities consistent with diastolic dysfunction or elevated filling pressures [1,2,3]. As stated in the latest guidelines from the European Society of Cardiology (ESC) and the American Heart Association (AHA) [2,4,5], several useful diagnostic algorithms have been proposed [6,7]. The diagnosis and prognostic assessment of this syndrome remain challenging due to its heterogeneity, complex pathophysiology, and the coexistence of multiple comorbidities. Patients are classified based on clinical, hemodynamic parameters, or biomarkers, often resulting in overlapping phenotypes [6,7,8]. HFpEF is, therefore, a multifactorial condition, with diastolic dysfunction [9], inflammation [3,10], and metabolic disturbances [11,12] playing central roles. Evidently, abnormalities are observed at the level of myocardial cells [13] and the extracellular matrix [14]. Phenotypic categorization of patients allows for more individualized and effective management. Six main HFpEF phenotypes are currently considered: (a) obesity-related, (b) atrial myopathy, (c) pulmonary hypertension (PH), (d) arterial stiffness with reduced aortic compliance, (e) ischemic phenotype (including both macrovascular and microvascular ischemia), and (f) diabetic phenotype [8]. Natriuretic peptides (NPs), though well established for HF risk stratification and therapeutic guidance [15,16], may be less useful in HFpEF compared to HF with reduced ejection fraction (HFrEF), as their levels are influenced by many variables such as age, obesity, and atrial fibrillation [17]. Beyond NPs, soluble suppression of tumorigenicity-2 (sST2) and galectin-3 (Gal-3) have emerged as important novel biomarkers, as they are associated with fibrosis and inflammation and may therefore play a critical role in the natural history of HF [9,18]. Plasma concentrations of sST2 and Gal-3 have been identified as prognostic indicators in heart failure; however, their diagnostic utility appears to be low [18]. These biomarkers correlate with NP levels, but they do not display the same limitations [19]. This scoping review aimed to evaluate the diagnostic and prognostic role of sST2 and Gal-3, as they appear to have fewer limitations compared to NPs in patients with HFpEF.

## 2. Methods

This scoping review was conducted to evaluate the role of the circulating biomarkers sST2 and Gal-3 in the management (diagnosis and risk stratification) of patients with HFpEF. The review was performed in accordance with the Preferred Reporting Items for Systematic Reviews and Meta-Analyses extension for Scoping Reviews (PRISMA-ScR). A literature search was conducted in the PubMed and ScienceDirect databases to identify relevant studies evaluating the association of sST2 and Gal-3 with HFpEF. The final search was performed on September 2025, and studies published between January 2018 and July 2025 were considered eligible. The search strategy included MeSH terms and free-text keywords using Boolean operators: ("Heart Failure"[MeSH] OR "heart failure with preserved ejection fraction" OR HFpEF) AND ((sST2 OR "suppression of tumorigenicity 2") OR ("galectin-3" OR "Galectin 3"[MeSH])). Titles and abstracts of retrieved records were screened to identify potentially relevant studies, followed by full-text assessment according to predefined eligibility criteria. Studies were included if they evaluated the diagnostic or prognostic significance of sST2 or Gal-3 in patients with HFpEF. Articles not focusing on HFpEF populations, studies not reporting clinical associations of these biomarkers, review articles, case reports, and editorials were excluded. Grey literature sources and clinical trial registries were not searched in the present review. Only studies published in English were considered eligible for inclusion, due to resource limitations, specifically the lack of access to translation services for non-English full-text articles. Although this may introduce some degree of language bias, it was considered necessary to ensure accurate screening and data extraction. Data from eligible studies were extracted and summarized in descriptive tables to provide an overview of study characteristics, patient populations, and the reported clinical implications of the investigated biomarkers. A review protocol was not prospectively developed or registered for this scoping review.

## 3. Results

### 3.1 Study Selection

Out of 4665 initial results, 119 articles were selected based on their titles. After removing duplicates, 83 articles remained. Exclusion criteria included meta-analyses, review articles, publications not written in English, studies that did not involve patients with HFpEF, and articles not relevant to the scope of this review. Ultimately, 11 studies were included, which investigated patients with HFpEF either as the main group or as a subgroup analysis. The study selection process is summarized in the PRISMA-ScR flow diagram (Fig. 1, Ref. [20]).

**Fig. 1:**
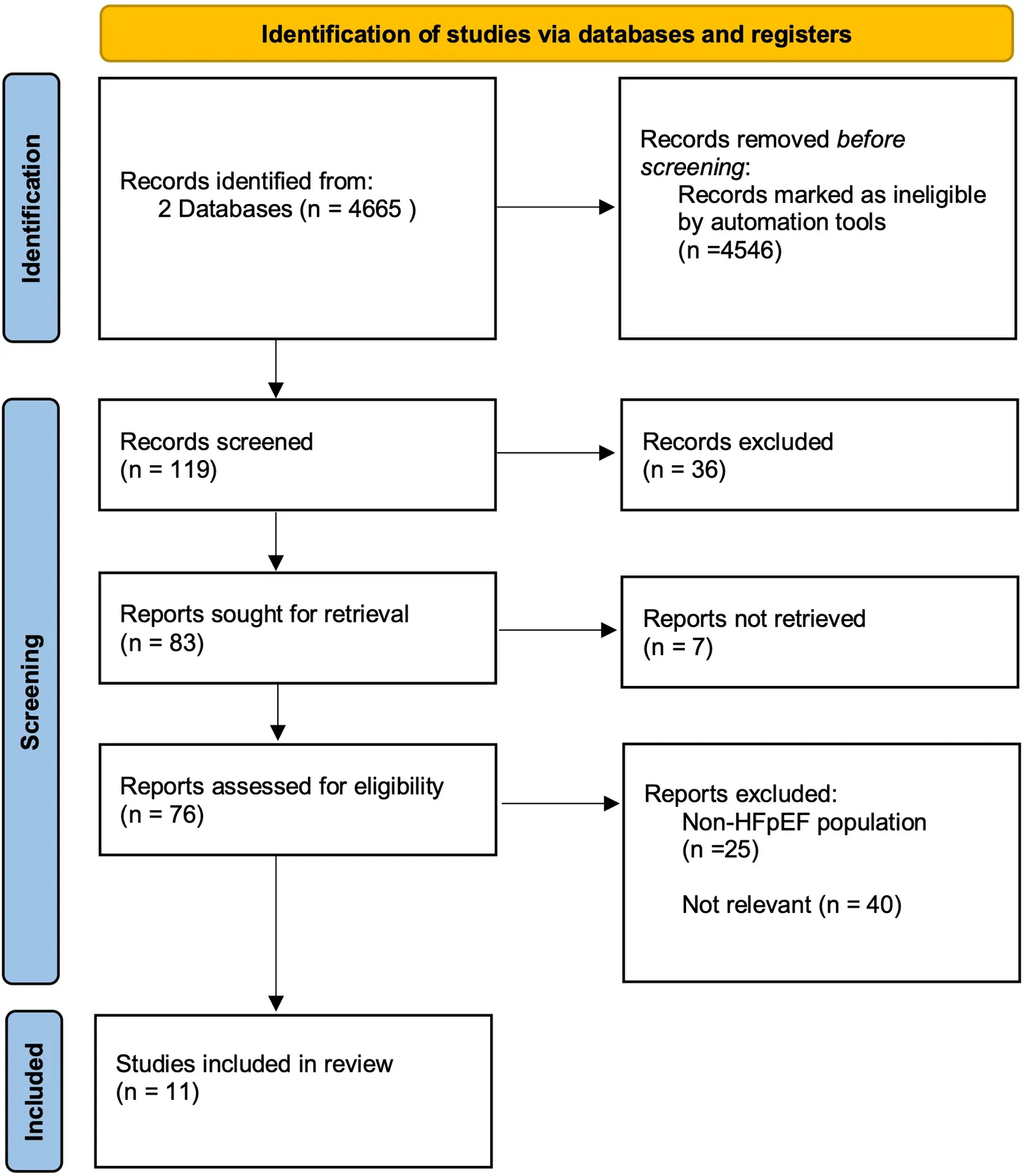
**Preferred Reporting Items for Systematic Reviews and Meta-Analyses extension for Scoping Reviews (PRISMA-ScR) flow chart [20]**.

### 3.2 Characteristics

PRISMA-ScR flow chart was used (Fig. 1) [20]. Table 1 (Ref. [18,21,22,23,24,25,26,27,28,29,30,31,32,33,34,35,36]) presents the specific characteristics of the studies selected based on the aforementioned criteria. Five studies investigated prognosis, four focused on diagnosis, whereas two examined both prognosis and diagnosis. Patients were included either upon admission for acute heart failure (initial diagnosis), decompensation of known chronic heart failure, or following evaluation in a cardiology department to which they had been referred. Notably, several studies, patients with autoimmune diseases—as well as those with conditions associated with significant inflammation [18,21,22,23,24] or malignancy [18,21,22,23,25]—were excluded.

**Table 1.** **Study characteristics and findings**.

Authors	Study design (Purpose)	Study location Sample size*	Mean follow-up (years)	Characteristics	Biomarker cut-off/Measurement technique (manufacturer)	HR and endpoints	Adjusted model	HR and endpoints	Adjusted model
Sugano et al. [26]	Prospective cohort (Prognosis)	Japan 191 (100%)	1.2	Acute HF pts	logsST2 ELISA (**)	1.02 (1.01–1.03), *p* < 0.001 for all-cause mortality	-	1.02 (1.00–1.03), *p* = 0.01 for CV death	-
Huang et al. [21]	Prospective cohort (Prognosis)	China 164 (45.73%)	1	Hospitalized pts	sST2 >117.80 ng/mL ELISA (**)	-	-	-	-
Cui et al. [22]	Prospective cohort (Prognosis and diagnosis)	China 247 (69.6%)	1	Acute HF pts	LogsST2 ELISA (unknown manufacturer)	1.34 (1.14–1.57), *p* = 0.089 for CV death or HF hospitalization	Model Α	1.29 (1.17–1.42), *p* = 0.156 for CV mortality or HF hospitalization	Model Α and NT-proBNP
Cui et al. [22]	-	-	-	-	LogGal-3 ELISA (***)	2.57 (2.18–2.84), *p* = 0.000 for CV death or HF hospitalization	Model A	2.33 (1.72–2.94), *p* = 0.009 for CV mortality or HF hospitalization	Model Α and NT-proBNP
Chen et al. [30]	Retrograde observational cohort (Prognosis)	China 1726 ^x^	1.9	Acute HF pts	sST2 >26 ng/mL^≠^ Fluorescence immunoassay (****)	1.3 (1.07–1.6), *p* = 0.01 for primary endpoint^××^	Age, Sex, DM, ΒΜΙ, HTN, ischemic cardiac disease, ΝΥΗΑ class, eGFR, NT-proBNP, hscTnT	1.28 (1.02–1.61), *p* = 0.033 for primary endpoint^××^	‡
Pan et al. [18]	Cross-sectional (Diagnosis)	China 113 (53.1%)	2.16	Hospitalized pts	sST2 >0.332 ng/mL ELISA kit No. EK1116 (⁕)	-	-	-	-
Song et al. [23]	Prospective observational cohort (Prognosis)	China 405 (27.16%)	1	Hospitalized pts	sST2 >56.89 ng/mL^†^ PRESAGE ELISA kit (⁕⁕)	6.48 (1.89–22.21), *p* = 0.003 for all‐cause mortality/HF hospitalization	Age, Sex, SAP, NYHA, class, smoking, CD, DM	7.07 (2.30–21.72), *p* = 0.001 for all‐cause mortality/HF hospitalization	-
Kanukurti et al. [28]	Cross-sectional (Diagnosis)	India 83 (75.9%)	-	Referral by cardiologist	Gal-3 >10.1 ng/mL ELISA (Elabscience Human ELISA kit, Houston, TX, USA)	-	-	-	-
Jirak et al. [24]	Retrospective cohort (Diagnosis)	Germany 252 (7.14%)	-	Stable pts	sST2 ^≠≠^ ELISA (**)	-	-	-	-
Emdin et al. [27]	Retrospective cohort (Prognosis) º	4268 (5%)	2.4	Chronic HF	log2sST2 Presage ELISA kit (Critical Diagnostics San Diego, CA, USA)	1.97 (1.21–3.21), *p* = 0.007 for all‐cause mortality	Model B	1.47 (1.02–2,14), *p* = 0.004 for HF hospitalization	Model B
Jiang et al. [25]	Observational cohort (Diagnosis)	China 192 (50%)	-	Hospitalized pts	Gal-3 >15.974 ng/mL ELISA (Unknown manufacturer)	-	-	-	-
Trippel et al. [29]	Prospective observational study (Diagnosis and prognosis)	Germany 1386 (7.72% at baseline)	10	Stable pts ºº	Log2(galectin‐3) ELISA (BG Medicine, Inc., Waltham, MA, USA)	2.16 (1.43–3.27), *p* < 0.001 for all‐cause mortality	-	1.87 (1.39–2.52), *p* < 0.001 for cardiovascular mortality or HF hospitalization	-

*Sample size (% HFpEF); ** R&D Systems, Minneapolis, MN, USA; *** Qiyi Biological Co., Shanghai, China; **** Boditech, Guangxi, China; ⁕Wuhan Boshide Biological Company; ⁕⁕ Critical Diagnostics, CA, USA; º Data were derived from six cohort studies: Barcelona cohort, Pro-Brain Natriuretic Peptide Outpatient Tailored CHF Therapy (PROTECT), Controlled Rosuvastatin Multinational Trial in Heart Failure (CORONA), Val-HeFT, Trial of Intensified versus Standard Medical Therapy in Elderly Patients with Congestive Heart Failure (TIME-CHF), VitD-CHF [31,32,33,34,35,36]; ^x^ the percentage of HFpEF subpopulation is not reported; ^××^ primary endpoint: cardiovascular death, or all-cause mortality/need for LVAD/heart transplantation; ^≠≠^ cut-off not reported; ^‡ ^Age, ΒΜΙ, atrial fibrillation, ΝΥΗΑ class, use of ACEi, ARB/ARNI, BB, levels of Hgb, albumin, potassium, sodium, eGFR, NT-proBNP, hscTnT, Model Α: Age, sex, EF, AP, NYHA class, CD, Hypertension, BB, MRA, LDL-C, eGFR; ^≠^ for acute HF 62 ng/mL was the cut-off; ^†^ third tertile (first <28.86 ng/mL, second 28.86–56.89 ng/mL); ºº Eligible pts were considered aged 50 to 85 years with ≥1 risk factor for HF: hypertension, diabetes mellitus, sleep apnoea, and atherosclerotic disease, or a history of HF, Model B: Age, Sex, HF etiology (ischemic or non-ischemic), EF, NYHA class I–II versus III–IV, eGFR, use of ACEi/ARB, BB, MRA, log2NT-proBNP, hs-cTnTI. ELISA, enzyme‐linked immunosorbent assay; HF, heart failure; sST2, soluble suppression of tumorigenicity-2; Gal-3, galectin-3; HR, hazard ratio; CV, cardiovascular; DM, diabetes mellitus; ΒΜΙ, body mass index; HTN, hypertension; ΝΥΗΑ, New York Heart Association; eGFR, estimated Glomerular filtration rate; NT-proBNP, N-terminal pro-Brain Natriuretic Peptide; SAP, systolic arterial pressure; CD, coronary disease; ACEi, angiotensin-converting enzyme inhibitors; ARB, angiotensin 2 receptor blockers; ARNI, angiotensin receptor/neprilysin inhibitor; BB, beta-blockers; hs-cTnTI, high-sensitivity cardiac troponin I; AP, arterial pressure; EF, ejection fraction; MRA, mineralocorticoid receptor antagonist; LDL-C, low-density lipoprotein cholesterol; Hgb, hemoglobin; pts, patients.

### 3.3 Biomarker Measurement

Enzyme‐linked immunosorbent assay (ELISA) was the most common method for the measurement of sST2 [18,21,22,23,24,26,27] and Gal-3 levels [22,25,28]. In total, five studies used samples from stable patients [24,26,27,28,29], and the rest of the studies collected samples 48 hours post-admission [18,21,22,23,25,30]. Marker levels were assessed in stored samples, mainly at a temperature of –80 ºC [21,22,23,24,27,29,30], −70 °C as reported by Pan et al. [18] and –40 °C according to Kanukurti et al. [28]. Specific details of the biomarker measurement are presented in Table 1. The diagnosis of HFpEF was established based on the current guidelines in effect at the time, and LVEF was evaluated either by visual estimation or the Simpson’s method by an experienced imagist. As this is a scoping review, assay methods and manufacturer details were reported as described in the original studies and were not standardized across included studies.

### 3.4 Outcome

The thresholds used to interpret biomarkers varied based on the measurement method and the characteristics of the population studied. In most studies, the hazard ratio (HR) is calculated using Cox regression analysis for not normally distributed variables.

1. sST2: According to Emdin et al. [27], 28 ng/mL was the optimal cut-off value for determining risk of HF hospitalization in stable patients. However, Huang et al. [21] reported a cut-off of 117.80 pg/mL, potentially due to a different measurement method as well as the different state of the study population (specifically, hospitalized patients were included). Concerning the diagnosis, Pan et al. [18] reported a threshold of 0.332 ng/mL, a decreased cut-off mainly due to a different ELISA kit (No. EK1116). Higher levels were described in HFrEF [18,21] whereas no statistically significant difference was observed between Heart Failure with mildly reduced Ejection Fraction (HFmrEF) and HFpEF patients [21]. These markedly lower values compared to other studies are attributable to differences in assay methodology and units, and therefore are not directly comparable. On the contrary, Song et al. [23] noted that the biomarker levels did not fluctuate across the spectrum of HF, acknowledging the inherent limitations and potential biases of a case series study. However, variability in study design, patient populations, and biomarker cut-off values across studies limits the direct comparability of findings.

2. Gal-3: [29]. This biomarker demonstrated higher diagnostic value in HFpEF compared to sST2, with a threshold of 9.55 ng/mL in patients with newly diagnosed or decompensated HFpEF [22]. Regarding stable patients, the optimal diagnostic threshold fluctuated from 10.1 ng/mL [28] to 13.57 ng/mL [29]. Of note, the mean Gal-3 level in a small cross-sectional case–control study was found to be particularly high at 26.59 ng/mL, perhaps due to population characteristics (19% of the cohort presented with chronic kidney disease) [28]. However, variability in study design, patient populations, and biomarker cut-off values across studies limits the direct comparability of findings.

### 3.5 Findings

The definitions of outcomes varied across the studies. The measures of association, *p*-values, and the specific comparisons examined in each study are presented in Table 1 and Table 2 (Ref. [18,21,22,24,25,28,29]). Of the eleven studies included, five investigated the relationship between sST2 levels and prognosis, with four reporting statistically significant results (*p*-value < 0.05). Three studies examined the association between sST2 levels and the diagnosis of HFpEF; one of these demonstrated a statistically significant correlation, while another did not document a *p*-value. Specifically, five studies assessed the relationship between sST2 levels and all-cause mortality or hospital readmission. Of these, Chen et al. [30], also evaluated the risk of heart transplantation or the need for left ventricular assist device (LVAD) implantation, adjusted for multiple clinical and laboratory parameters, and observed statistically significant associations. Four studies assessed the diagnostic utility of Gal-3, all of which reported statistically significant results (*p* < 0.05); in addition, two of these studies also demonstrated prognostic relevance. The study by Sugano et al. [26] highlights the significance of sST2 for non-cardiovascular mortality (mainly due to infection or sepsis). sST2 levels were associated with all-cause mortality as well as non-cardiovascular death, with a reported relative risk (RR) of 1.02 (1.01–1.03), *p*-value < 0.001, and 1.03 (1.01–1.05), *p*-value = 0.002, respectively (with similar results after adjustment for age and sex). According to Huang et al. [21], sST2 had a strong negative predictive value (83.3%) for cardiovascular death or hospitalization due to HF during one-year follow-up, despite lower values in the HFpEF population. In conclusion, sST2 serves as an important predictor of cardiovascular mortality and HF hospitalizations, as a standalone measure and complementary to N-terminal pro-Brain Natriuretic Peptide (NT-proBNP).

**Table 2.** **Diagnostic and prognostic value of sST2 and Gal-3**.

	Specificity	Sensitivity	AUC	Marker’s cut-off limit	*p*-value
Cui et al. [22] Gal-3, diagnostic marker	86%	65%	0.819 (0.75–0.89)	9.55 ng/mL	0.000
Cui et al., [22] sST2, diagnostic marker	57%	48%	0.584 (0.49–0.68)	68.6 pg/mL	0.17
Jiang et al. [25] Gal-3, diagnostic marker	71.9%	76%	0.763 (0.696–0.821)	15.974 ng/mL	<0.0001
Pan et al. [18] sST2, diagnostic marker	95%	51.7%	0.717 (0.628–0.796)	0.332 ng/mL	<0.01
Kanukurti et al. [28] Gal-3, diagnostic marker	95%	77.78%	0.927	10.1 ng/mL	<0.001
Jirak et al. [24] sST2, diagnostic marker	-	-	0.567 (0.294–0.572)	-	-*
Huang et al. [21] sST2, prognostic marker for CV death or HF hospitalization	64.3%	83.3%	0.677 (0.492–0.862)	117 ng/mL	0.042
Trippel et al. [29] Gal-3, diagnostic marker	73%	61%	0.71	13.57 ng/mL	<0.001

* The DeLong test was conducted to compare the Area Under the Curve (AUC) values of various markers, including sST2.

Cui et al. [22] compared Gal-3 and sST2, finding Gal-3 to be superior to sST2 in differentiating HFpEF from the control population; nevertheless, both markers showed significant associations with major cardiovascular events. The study by Chen et al. [30] indicated that in hospitalized HFpEF patients, log sST2 was independently associated with major endpoints (death, heart transplantation, or need for LVAD) in a univariate Cox regression analysis [HR 1.78, 95% CI (1.50, 2.11), *p* < 0.001] with a mean follow-up duration of about two years. The same study also examined the longitudinal value of sST2 for predicting the primary endpoint, using different thresholds of 25.1, 27.1 and 27.2 ng/mL with satisfactory Area Under the Curve (AUC) values. A key limitation of this study, though, is selection bias, as patients without an sST2 measurement on admission were excluded. In addition, patients with HF and sST2 >0.332 ng/mL have approximately a six times higher mortality rate, according to Pan et al. [18], who also demonstrated that NT-proBNP offers a higher diagnostic value. In line with these findings, Song et al. [23] showed that higher sST2 values are associated with more severe clinical presentation (New York Heart Association (NYHA) III/IV) and thus, these patients exhibit a higher mortality risk irrespective of the ejection fraction. Interestingly, the combined use of sST2 and NT-proBNP has greater prognostic value than NT-proBNP alone [23].

In the study by Kanukurti et al. [28], Gal-3 was shown to be a valuable diagnostic marker for HFpEF, with improved accuracy when combined with NT-proBNP. Specifically, Gal-3 was assessed using a threshold of 10.1 ng/mL, yielding a specificity of 95% and a positive predictive value of 98% (*p* < 0.001). Similarly, a multicentre study on the Prevalence and Clinical Course of Diastolic Dysfunction and Diastolic Heart Failure (Diast‐CHF) [29] showed that higher Gal-3 concentrations were significantly associated with an increased risk of developing HFpEF, higher adjusted all-cause mortality, and a greater risk of cardiovascular hospitalization or death. The diagnostic utility of Gal-3 was also found to be superior to that of NT-proBNP. Of note, this group was non-randomized and consisted of patients with cardiovascular risk factors [29].

In the study by Jirak et al. [24], four novel biomarkers were compared. Among them, sST2 appeared to have inferior diagnostic value over Growth Differentiation Factor (GDF-15) for HFpEF. However, several limitations should be acknowledged, including the small sample size of the HFpEF cohort (18 patients) and the absence of diagnostic cut-off values for each biomarker, as the primary aim of the study was to compare their relative diagnostic performance. In the study by Emdin et al. [27], a single measurement of sST2 provided prognostic value for mortality or risk of hospitalization due to heart failure, irrespective of age, sex, etiology of heart failure, LVEF, renal function, or levels of NT-proBNP and high sensitivity Troponin T (hs-TnT). Finally, the study by Jiang et al. [25] demonstrated that although Gal-3 levels were markedly elevated in HFpEF, the diagnostic accuracy was moderate. However, Gal-3 outperformed individual echocardiographic parameters, underscoring its potential clinical relevance in unmasking diastolic dysfunction.

## 4. Discussion

An effective biomarker should be cost-efficient, easy to use, interpretable, and involved in disease pathophysiology [15]. Currently, NPs remain the gold standard biomarkers for heart failure, as highlighted by the STRONG-HF and Pro-Brain Natriuretic Peptide Outpatient Tailored CHF Therapy (PROTECT) studies, although their prognostic value is lower in HFpEF than in HFrEF [36,37,38]. In HFpEF, NP levels may be affected by lower wall stress, atrial fibrillation, left atrial remodeling, age, renal function, inflammation, obesity, and ethnicity, potentially leading to underdiagnosis and underestimated prognosis [39,40,41,42]. A theory of endogenous NP deficiency is also proposed [43]. Novel biomarkers, particularly sST2 and Gal-3, show promise in overcoming these challenges. sST2 is produced by alveolar (type II pneumocytes), endothelial cells, fibroblasts, and myocardial cells. Its role involves opposing the cardioprotective interleukin-33/suppression of tumorigenicity-2 (IL-33/ST2) pathway, which inhibits fibrosis, hypertrophy, and myocardial cell apoptosis [16,44,45,46,47]. Nonetheless, its specificity is relatively limited because sST2 is not exclusively cardiac-specific. Increased levels appear in extra-cardiac conditions such as systemic inflammation and pulmonary disease. However, sST2 is secreted in response to mechanical stress or myocardial injury, correlates with pulmonary congestion and increased afterload, which are key mechanisms in HFpEF [48,49,50]. The most validated measurement method is the “Presage ELISA” immunoassay, and the optimal prognostic threshold for sST2 is debated; a value of 32–35 ng/mL is generally cited for risk stratification in HF patients [46,51]. Several studies demonstrate that sST2 is an independent and additive prognostic factor in HF, predicting mortality, cardiovascular death, and heart failure hospitalizations, particularly in patients with preserved EF, and in cases where NT-proBNP levels are reduced [45,49,50,52]. Unlike NPs, sST2 appears advantageous as it is not influenced by age, obesity, renal function, or race [39,53]. The correlation between these two biomarkers is not strong [26,27,54], reflecting different biological pathways and clinical implications [49,55].

Gal-3 is a β-galactoside-binding lectin expressed in many cells, including activated cardiac macrophages, contributing to the cascade of fibrosis and inflammation [56,57]. It is being studied for its role in remodeling, in diastolic dysfunction [58,59] and has emerged as a diagnostic biomarker in HFpEF [60]. There is limited data regarding Gal-3 levels and therapeutic response in HFpEF. Like sST2, Gal-3 is not influenced by obesity or race (although it increases with age and renal impairment). Studies such as Controlled Rosuvastatin Multinational Trial in Heart Failure (CORONA) [61] and a Gal-3 substudy of N-terminal Pro-BNP Investigation of Dyspnea in the Emergency Department (PRIDE) [62,63,64] suggest that Gal-3 may be a stronger prognostic factor than NT-proBNP for risk stratification.

As elevated plasma concentrations of sST2 and Gal-3 are not cardiac-specific, these biomarkers have limited diagnostic value. Research interest has therefore focused on their potential prognostic information in heart failure patients, both independently and in addition to established markers such as cardiac troponins or NPs. Most researchers show improved prognostic performance when combining NPs with these newer biomarkers [23,26,65], while acknowledging their limitations and potential bias. Studies included in our review demonstrated the prognostic value of sST2 for mortality, both individually and in combination with NPs. Fewer studies examined Gal-3, which revealed strong prognostic but moderate diagnostic value. Differences in prognostic and diagnostic thresholds for these biomarkers are possibly due to variations in sampling and measurement methods. Indeed, sampling occurred within 48 hours of admission in six studies [18,21,22,23,25,30], whereas in four studies, samples were collected from stable patients [24,26,27,28]. Biomarker measurement was mainly performed with the method of ELISA, but the assay manufacturer and storage temperature varied. The criteria for defining the study populations were not entirely homogeneous: they generally followed current guideline recommendations, except for the studies by Sugano et al. [26] (diagnosis of HF was established using the Framingham criteria) and Emdin et al. [27] (in Trial of Intensified versus Standard Medical Therapy in Elderly Patients with Congestive Heart Failure (TIME-CHF) population diagnosis was based on NP values: NT-proBNP ≥400 ng/L for ≥60 years or ≥800 ng/L for ≥75 years). Regarding sample definition, Ejection Fraction (EF) was assessed by Simpson’s method or visually, with possible interobserver variability. Endpoints also varied: mortality, HF hospitalization, and (in one study) need for LVAD [30]. Limitations of these studies include systematic error due to small sample sizes and selection bias (excluded populations without sampling in many studies).

We are still far from clinical application of these biomarkers. A reliable measurement method and a defined threshold must be set for comparable and trustworthy results. In nearly all published studies on heart failure patients, plasma concentrations of sST2 and Gal-3 have been measured using immunoassays, some of which can be fully automated [39]. However, results from different methods are not directly comparable. For sST2, substantial method-dependent variation has been observed; to a lesser extent, the same applies to Gal-3. The intra-individual variability of these biomarkers over time has also been assessed, which is important for interpreting multiple consecutive measurements. In one of the largest related studies, sST2 and Gal-3 showed low variability [66]. Although many factors complicate the clinical use of these biomarkers (different detection techniques and biological parameters, such as age, sex, and clinical context: acute vs. chronic heart failure), sST2 and Gal-3 were found to be stable and potentially reliable for clinical application [39,66]. Thus, a multimarker assay with sST2, Gal-3 and NT-proBNP should be developed and validated for clinical application through large carefully designed multicenter studies. Future research should focus on the integration of multimarker approaches, including Galectin-3 and sST2, to improve risk stratification in HFpEF. Additionally, standardization of assay methodologies is essential to enhance comparability across studies. Prospective studies are needed to further validate the clinical utility of these biomarkers and their role in guiding therapeutic decision-making.

## 5. Conclusions

Heart failure with preserved ejection fraction remains a complex clinical syndrome with significant diagnostic and prognostic challenges. Current evidence from moderate-quality observational studies suggests that novel biomarkers such as sST2 and Gal-3 have potential prognostic utility in HFpEF, particularly through their associations with myocardial fibrosis, inflammation, and disease severity. These biomarkers reflect important pathophysiological pathways involved in HFpEF and may contribute to improved risk stratification and patient phenotyping beyond conventional NPs. Nevertheless, the available evidence remains limited due to heterogeneous study populations, small sample sizes, variable biomarker cut-off values and the lack of standardized assays, thus hindering broader clinical implementation. Future research should focus on validating their prognostic value through large-scale prospective studies and exploring their integration into uniform multimarker protocols in HFpEF patients.

## Abbreviations

AHA, American Heart Association; AUC, Area Under the Curve; CI, Confidence Interval; CORONA trial, Controlled Rosuvastatin Multinational Trial in Heart Failure; Diast-CHF, Prevalence and Clinical Course of Diastolic Dysfunction and Diastolic Heart Failure; EF, Ejection Fraction; ELISA, Enzyme-linked Immunosorbent Assay; ESC, European Society of Cardiology; Gal-3, Galectin-3; GDF-15, Growth Differentiation Factor-15; HF, Heart Failure; HFmrEF, Heart Failure with mildly reduced Ejection Fraction; HFpEF, Heart Failure with preserved Ejection Fraction; HFrEF, Heart Failure with reduced Ejection Fraction; HR, Hazard Ratio; hs-TnT, High-sensitivity Troponin T; IL-33/ST2, Interleukin-33/Suppression of Tumorigenicity-2; LAVi, Left Atrial Volume Index; LVEF, Left Ventricular Ejection Fraction; LVAD, Left Ventricular Assist Device; NPs, Natriuretic Peptides; NT-proBNP, N-terminal pro-Brain Natriuretic Peptide; NYHA, New York Heart Association; PH, Pulmonary Hypertension; PRIDE study, N-terminal Pro-BNP Investigation of Dyspnea in the Emergency Department; PROTECT study, Pro-Brain Natriuretic Peptide Outpatient Tailored CHF Therapy Study; RR, Relative Risk; sST2, Soluble Suppression of Tumorigenicity-2; TIME-CHF trial, Trial of Intensified versus Standard Medical Therapy in Elderly Patients with Congestive Heart Failure.
